# Improper excess light energy dissipation in *Arabidopsis *results in a metabolic reprogramming

**DOI:** 10.1186/1471-2229-9-12

**Published:** 2009-01-26

**Authors:** Martin Frenkel, Carsten Külheim, Hanna Johansson Jänkänpää, Oskar Skogström, Luca Dall'Osto, Jon Ågren, Roberto Bassi, Thomas Moritz, Jon Moen, Stefan Jansson

**Affiliations:** 1 Umeå Plant Science Centre, Department of Plant Physiology, Umeå University, SE-901 87 Umeå, Sweden; 2 Department of Ecology and Environmental Science, Umeå University, SE-901 87 Umeå, Sweden; 3 Dipartimento Scientifico e Tecnologico, Università di Verona, Strada Le Grazie, 15- 37134 Verona, Italy; 4 Department of Ecology and Evolution, EBC, Uppsala University, Villavägen 14. SE-752 36 Uppsala, Sweden; 5 Université Aix-Marseille II, LGBP- Faculté des Sciences de Luminy, Département de Biologie, Case 901, 163, Avenue de Luminy, 13288 Marseille, France; 6 Umeå Plant Science Centre, Department of Forest Genetics and Plant Physiology, Swedish University of Agricultural Sciences, SE-901 87 Umeå, Sweden

## Abstract

**Background:**

Plant performance is affected by the level of expression of PsbS, a key photoprotective protein involved in the process of feedback de-excitation (FDE), or the qE component of non-photochemical quenching, NPQ.

**Results:**

In studies presented here, under constant laboratory conditions the metabolite profiles of leaves of wild-type *Arabidopsis thaliana *and plants lacking or overexpressing PsbS were very similar, but under natural conditions their differences in levels of PsbS expression were associated with major changes in metabolite profiles. Some carbohydrates and amino acids differed ten-fold in abundance between PsbS-lacking mutants and over-expressers, with wild-type plants having intermediate amounts, showing that a metabolic shift had occurred. The transcriptomes of the genotypes also varied under field conditions, and the genes induced in plants lacking PsbS were similar to those reportedly induced in plants exposed to ozone stress or treated with methyl jasmonate (MeJA). Genes involved in the biosynthesis of JA were up-regulated, and enzymes involved in this pathway accumulated. JA levels in the undamaged leaves of field-grown plants did not differ between wild-type and PsbS-lacking mutants, but they were higher in the mutants when they were exposed to herbivory.

**Conclusion:**

These findings suggest that lack of FDE results in increased photooxidative stress in the chloroplasts of *Arabidopsis *plants grown in the field, which elicits a response at the transcriptome level, causing a redirection of metabolism from growth towards defence that resembles a MeJA/JA response.

## Background

The photosynthetic light reaction provides plants with the energy required for growth and metabolism. However, due to the inherent properties of the excited pigment molecules and high-energy electron carriers, harmful side reactions can occur that can lead to the destruction of pigments, proteins and lipids and, unless these side reactions are controlled, death of the plant. Several mechanisms for regulating the photosynthetic light reaction have evolved, and the fact that all photosynthetic organisms have photoprotective processes, operating in different ways and over varying timescales, suggests that they are essential for survival. Feedback de-excitation (FDE), also called the qE – or energy-dependent – component of non-photochemical quenching (NPQ), is a photoprotective process of crucial importance for the plant. PsbS, a 22 kDa protein of the LHC superfamily, is necessary for the functioning of FDE [1]. Shortly after an increase in light intensity, plants reduce the excitation pressure on photosystem II (PSII) by inducing FDE, changing the conformation of the photosynthetic antenna from an "energy transfer state" to a "quenched state", allowing excess energy to be dissipated as heat. Feedback de-excitation is reversible within a few minutes, whereas state transition, a second mechanism that can reduce excitation pressure, is reversible over slightly longer time-scales (ca. 15 minutes). Plants lacking FDE through mutations in the PsbS gene show no marked phenotypic deviations from their respective wild-type in controlled growth chamber conditions [1,2], but exhibit reductions in fitness when grown under fluctuating light or field conditions [3]. Other short-term responses to increases in light intensities include increases in cyclic electron transfer rates, activation of the Calvin cycle, and photorespiration. Long-term responses include reductions in effective light intensities by thickening and tilting of leaves, accumulation of anthocyanins, and movements of the chloroplasts [reviewed by: [4]]. A reduction in light harvesting can be achieved by reducing the size of the effective light-harvesting antenna, while energy utilization can be increased by up-regulation of the "dark" reactions. In addition, plants have scavenging systems that neutralize light-induced reactive oxygen species (ROS). ROS are generated, to some degree, at all light intensities, but especially when the intensity exceeds the capacity of the plant's photon utilization mechanisms.

Previously we have attempted to elucidate the mechanistic background of the reduced Darwinian fitness seen in field-grown plants lacking FDE by measuring selected physiological and metabolic parameters [5]. In the study reported here we have used DNA microarrays and metabolic profiling to obtain more information on ways in which plants respond to variations in FDE capacity, using mutants with no or overexpressed levels of PsbS. Furthermore, we have examined the responses of genotypes varying in PsbS expression, both in the field and laboratory, to the effects of herbivory. Since plants grown under natural conditions show larger plant-to-plant variation than those grown under controlled conditions a tightly controlled experimental design was used in order to obtain reliable data. If the amount of PsbS in wild-type plants is sufficient to saturate relevant responses, PsbS over-expressers would be expected to have an identical phenotype to that of wild-type plants (unlike PsbS-deficient plants). However, it is also possible that increasing FDE capacity to levels seen in the PsbS over-expressers may be beneficial in some respects. Thus, comparing the three types of plants in the field was expected to give information that could help assess this possibility, and provide further insights into plant responses affected by differences in FDE capacity. Here we present data that provide new indications regarding the role of FDE at the whole plant level, including evidence of an overlap between the stress responses resulting from increased excitation of photosystem II and those induced by the jasmonate pathway.

## Results

### PsbS overexpression did not have a significant impact on fitness

*Arabidopsis thaliana *genotypes *Col-0 *and oePsbSwere grown in an experimental garden during the summer of 2003, according to methods described by [3]. The weather was drier and warmer, the overall seed yield of wild-type plants was lower and their mortality rates were higher – suggesting that the plants were under greater stress – during 2003 than during the cited study (2000 and 2001. Measurements on field-grown plants confirmed that the two genotypes had FDE levels corresponding to expectations based on their PsbS levels (data not shown). OePsbS plants produced significantly more siliques, but fewer seeds per silique, than wild-type plants and consequently there was no significant difference in total seed production per plant between the two genotypes; oePsbS plants producing on average 0.3 (± 8.3)% more seeds than wild type seed plants. In contrast, *npq4 *plants produced on average 36% less seeds than wild type plants in 2000/2001 [3] and 2005 [6].

### PsbS levels influence plant metabolism under field conditions but not under standard lab conditions

**Figure 1:**
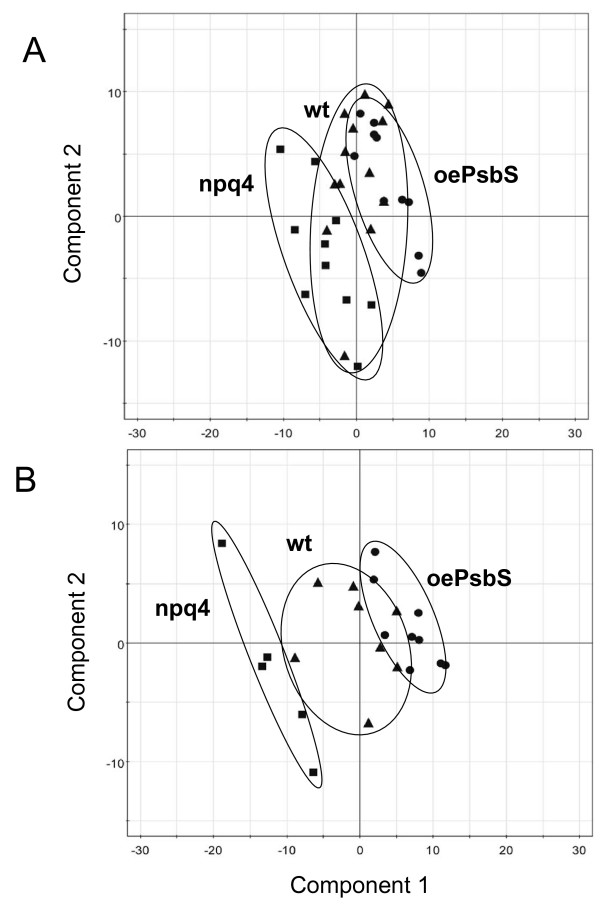
**PLS-DA analysis of metabolites**. PLS-DA analysis of the complete dataset obtained from the metabolomic analyses using GC-MS of leaves of *npq4 *(■), wild-type (▲) and oePsbS (●) *Arabidopsis *plants, grown under (A) controlled conditions and (B) in the field. All metabolite variables were log_10_-transformed, centred and scaled to unit variance prior to analysis. The PLS-DA score-plot shows the first two components, which were based on the oePsbS and *npq4 *sample-sets. Predictions were made for the wild-type data using the corresponding PLS-DA model. (A) 2 components: R^2^X = 0.19; R^2^Y = 0.89; Q^2^Y = -0.17. (B) 2 components: R^2^X = 0.38; R^2^Y = 0.97; Q^2^Y = 0.70.

Metabolic profiling by GC-MS was used to examine whether changes in PsbS expression had significant effects on plant metabolism. Leaves were sampled from at least 12 individuals of each genotype and their metabolomes were visualized by extracting the metabolites and analysing them by GC-MS, after derivatization of the samples, according to [7]. The extraction or GC-MS analysis of some biological replicates failed for various reasons, so they were excluded from further consideration. The remaining GC-MS data were analysed using hierarchical multivariate curve resolution (H-MCR; [8], and 357 components (putative metabolites) were resolved. The data were log10-transformed, centred and scaled to unit variance then subjected to partial least squares discriminant analysis (PLS-DA). PLS-DA analysis of the metabolite data from the three genotypes grown under constant light in the growth chamber did not result in any robust model, indicating that the metabolic profiles of the genotypes were not sufficiently different to distinguish between the respective genotypes (data not shown). PLS-DA based solely on the data obtained from *npq4 *and oePsbS plants grown in the lab resulted in a model with poor quality parameters (A and B) that weakly separated the genotypes, and provided predictions for the wild-type data that were not significantly different from those of either of the other two genotypes. The ordination plot shown in Fig. 1A illustrates the failure to distinguish between the three genotypes using the metabolic data acquired when the plants were grown under constant conditions.

In our earlier experiments [3] the fitness of the *npq4 *mutant was found to be similar to that of wild-type plants under constant environmental conditions. Differences only occurred when plants were grown under variable conditions, with the most notable effects arising in the field where they were exposed to irregular and unpredictable changes in, for instance, light intensity. Therefore, we performed a detailed metabolomic investigation of wild-type, *npq4 *and oePsbS plants grown under natural conditions in a similar manner to that described above. We also used DNA microarrays to document the total pattern of gene expression for the three genotypes.

Metabolic profiling of leaves from field-grown plants was performed in the same way as for plants grown under controlled conditions. As before, PLS-DA was used in an attempt to classify the three genotypes, and the first two components from the resulting model (R2X = 0.37, R2Y = 0.89, Q2Y = 0.19) successfully separated the genotypes (data not shown). To validate these results another PLS-DA model was calculated, using only the *npq4 *and oePsbS sample-sets, and predictions were made for the wild-type data based on this model. This model predicted that the wild-type plants would be located between the *npq4 *and oePsbS genotypes in most respects (Fig. 1B). The new model showed that the main differences between the genotypes appeared to be trends relating to PsbS expression, with *npq4 *and oePsbS at opposite ends and the wild-type in the middle, strongly implying that PsbS levels influence the composition of the plant metabolome.

Thus, there were distinct between-genotype differences in the metabolic profiles of the plants grown in the field, but not in those of plants grown under controlled conditions in the lab, even though variations between replicates were greater among plants grown in the field (Fig. 1).

### Major changes occurred in amino acid and carbohydrate metabolism

Following the observation of these effects, we attempted to identify metabolites whose concentrations were influenced by differences in PsbS levels. Comparison of the *npq4 *and oePsbS data revealed that levels of about 120 variables differed (i.e. the relative concentrations of the putative metabolites differed in plants of the two genotypes). Significant differences were identified by interpreting the loadings, [as described in [9]], obtained from the PLS-DA model (shown in Fig. 1B), and calculating 95% confidence intervals using jack-knifing (see, for instance, [10]). The significantly differing metabolites were then identified by comparing our retention indices and mass spectra with those in retention index and mass spectra libraries [11]. Mass spectra library searches resulted in the identification or classification of 45 compounds (Table 1 and, for a full list of compounds responsible for the separation of the genotypes, see Additional file 1). Calculations of the relative metabolite levels showed that in all except two cases (galactinol and GABA) the values for wild-type were between those of the *npq4 *and oePsbS plants. Ten of these metabolites were amino acids, three of which (leucine, tyrosine and threonic acid) were most abundant in *npq4*, whereas the others were highest in the oePsbS plants. Some of the differences were very pronounced; for example the glutamine concentration was seven times higher in oePsbS than in *npq4 *plants. Levels of β-alanine, an important amino acid required for biosynthesis of pantothenic acid (vitamin B5, which is required in turn for coenzyme A biosynthesis), were also higher in the oePsbS than in *npq4 *plants. Not only is β-alanine an important precursor in coenzyme A synthesis, but there have been suggestions that, along with pantothenate levels, it is involved in thermotolerance mechanisms in tobacco [12].

**Table 1:** Metabolites with significantly differing levels in wild-type, *npq4 *and oePsbS

Group	Metabolite	*npq4*	wt	oePsbS
Amino acids	Leucine	1.52	1.00	0.48
	Tyrosine	1.02	1.00	0.69
	Threonic acid	1.20	1.00	0.92
	β-Alanine	0.86	1.00	1.35
	Pyroglutamate	0.76	1.00	1.39
	Serine	0.78	1.00	1.72
	Aspartate	0.70	1.00	1.85
	Glycine	0.49	1.00	1.32
	Threonine	0.65	1.00	1.82
	Glutamine	0.47	1.00	3.33
	GABA	0.79	1.00	0.34

Carbohydr.	Galactose	2.00	1.00	0.06
	Raffinose	1.61	1.00	0.18
	Isomaltose	1.82	1.00	0.22
	Fructose	1.10	1.00	0.49
	Glucose	1.14	1.00	0.57
	Sorbose	1.10	1.00	0.58
	Ribose	1.27	1.00	0.77
	Arabitol	1.12	1.00	0.79
	1,6-Anhydro-β-D-glucose	1.26	1.00	0.90
	Unknown hexose	1.03	1.00	0.74
	Maltose	1.19	1.00	0.87
	Xylose	1.35	1.00	1.01
	Sucrose	0.86	1.00	1.02

Lipids/fatty a.	Digalactosylglycerol*	1.59	1.00	0.48
	Monogalactosylglycerol*	1.37	1.00	0.56
	Unknown sterol	0.93	1.00	1.09
	Glycerol-3-phosphate	0.92	1.00	1.18
	Stearic acid	0.89	1.00	1.14
	Fatty acid	0.91	1.00	1.18
	Glycerol	0.87	1.00	1.19
	Fatty acid	0.87	1.00	1.20
	α-Linolenic acid	0.98	1.00	1.54
	Linolenic acid	0.72	1.00	1.22
	Galactinol	0.88	1.00	0.12

TCA-cycle etcmyo-**Inositol**-1-phosph.		1.37	1.00	0.26
	Alpha-tocopherol	1.69	1.00	0.86
	Dehydroascorb. acid*	1.33	1.00	0.92
	Succinic acid	1.14	1.00	0.89
	Malic acid	0.98	1.00	1.11
	Fumaric acid	0.91	1.00	1.16
	Citrate	0.94	1.00	1.41
	Citramalic acid	0.84	1.00	1.18
	Spermidine	0.81	1.00	1.35

Values shown are relative amounts (per g FW) with wild-type levels set to 1. Significantly differing metabolites were detected by interpreting the first weight vector (w1), as described by [9], from the *npq4*/oePsbS PLS-DA model, together with the 95% confidence intervals calculated using jack-knifing [10]. Metabolites were analyzed as methyloxime-trimethylsilyl derivatives. Tentatively identified*

Similarly, carbohydrate metabolism was strongly influenced. Levels of galactose, raffinose and isomaltose were 30-, 9- and 8-fold higher, respectively in *npq4 *than in oePsbS plants. The levels of fructose and glucose also differed in a similar way, but not as markedly. Sucrose was the only carbohydrate detected that was more abundant in oePsbS than in *npq4 *plants. The trends in the amounts of glucose, fructose and sucrose between *npq4 *and wild-type were similar to those observed in plants grown in the field since germination, as detected by traditional metabolite analyses [5]. These findings indicate that plants grown in climate chambers and transferred to the field after five days have strong metabolic similarities, at least in their carbohydrate metabolism, to those grown in the field since germination.

Major changes in composition were also detected amongst lipid metabolites. For example, the *fatty *acids diacylgalactosylglycerol and monogalactosyl-glycerol were more abundant in *npq4*, while concentrations of certain other lipids were higher in oePsbS plants. However, it should be noted that our metabolite profiling approach is not optimized for analysing non-polar compounds, and specific changes in lipids should be analyzed by dedicated lipid analysis techniques. Notable differences detected in the abundance of metabolites of other classes included findings that concentrations of two compounds involved in detoxification of ROS, alpha-tocopherol and dehydroascorbic acid (tentatively identified), were higher in the *npq4 *plants, while other metabolites with central roles in primary metabolism, such as malate, fumarate and citrate, were more abundant in oePsbS, as was spermidine.

Overall, these data show that the "core metabolism" of *Arabidopsis*, as well as some of its secondary metabolism, responded quite dramatically to variations in levels of PsbS in plants grown in the field.

### PsbS levels influenced the transcriptome

**Figure 2:**
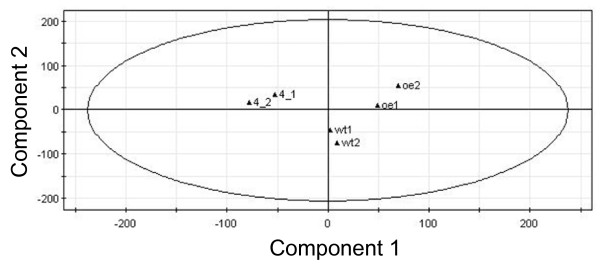
**Separation of *Arabidopsis *genotypes from gene expression profiles**. PCA score plot showing the complete dataset obtained from the transcriptomics analyses using DNA microarrays of rosette leaves of plants grown in the field of oePsbS (oe), wild-type (wt) and *npq4 *(4) *Arabidopsis *genotypes. The first two components from the PCA analysis is shown, 1 and 2 denote the two replicates of each genotype, each replicate containing RNA from a pool of plant individuals.

Prompted by the observed impact of PsbS levels on *Arabidopsis *primary metabolism, we next analyzed differences in the transcriptome of the three genotypes, in the expectation that they would provide further insights into the secondary effects of changes in FDE capacity and potentially help to elucidate the signal transduction pathway from the site of PsbS action (photosystem II). To do this we used a cDNA microarray approach (using CATMA microarrays) to measure global RNA expression in leaves of the three genotypes (*npq4*, wild-type and oePsbS) as grown in the field. The dataset is provided in Additional file 2. Initially, the data were compared using principal component analysis (PCA), an unsupervised ordination method. The three genotypes clearly separated from each other (Fig. 2), confirming that our experimental approach and experimental procedures had sufficient analytical power for drawing valid conclusions on transcriptomal differences between the three genotypes.

When comparing three samples, three different pair-wise comparisons can be made or one can attempt to integrate data from all samples into a single analysis. There were strong ordinal relationships between PsbS expression and the concentrations of various metabolites, with the wild-type in an intermediate position between *npq4 *and oePsbS along the x-axis, and similar patterns were observed in the transcriptome data (Fig. 2). Therefore, in the following section we will mainly discuss the transcriptomal differences observed between *npq4 *and wild-type plants, since the same general trends were found in the other pair-wise comparisons.

Out of the 25392 spots on the full CATMA cDNA array, expression data were obtained from 15167 (the remaining gave too weak hybridization signals), with 526 showing statistically significant differential expression (False Discovery Rate, FDR, corrected p < 0.05) between *npq4 *and oePsbS. The 23 genes that had the most significantly different expression levels are listed in Additional file 3. The increased level of mRNA encoding galactinol synthase, which catalyzes the first committed step in the synthesis of raffinose-family oligosaccharides (RFOs) [13], in *npq4 *plants is consistent with the high accumulation of raffinose in this genotype. Other genes that were significantly up-regulated in *npq4 *include, inter alia, those encoding a dormancy-associated protein, a lipoxygenase, adenylosuccinate lyase (which is involved in purine biosynthesis), arginase (which catalyzes the conversion of arginine to urea and ornithine), and 1,4-alpha-D-glucan maltohydrolase (beta-amylase).

### Expression levels of photosynthesis-related genes were higher in oePsbS

The development of Gene Ontology (GO) classifications and the introduction of MAPMAN software [14,15] have made it easier to study coordinated gene responses involved in specific biochemical pathways or physiological processes. Here, MAPMAN was used to visualize changes in transcription, since we believe it provides the most useful interface and allows more readily comprehensible classifications than other more complex categorization methods.

MAPMAN software includes a list of "BINs", which contain genes that have similar functions. For example, BIN 1 contains all nuclear genes involved in photosynthesis, and BIN 1.1 contains those encoding products involved in the photosynthetic light reactions. MAPMAN can graphically display the results from a microarray experiment, illustrating the overall expression trends of genes from specific BINs, and apply statistical tools to determine the BINs and subBINs that have been significantly up- or downregulated. When classes of genes represented by BINs are analyzed quality filtering can be applied less stringently than when individual differentially expressed genes are analyzed, because a single unregulated gene may show differential expression purely through chance when large numbers of genes are analysed, as they are in DNA microarray experiments. However, these events are stochastic, and analysis of a class containing several genes is less likely to show a false positive result. Hence, if the filtering is too stringent it may result in the failure to detect classes of genes that, for example, are all only slightly down-regulated, but appropriate statistical methods can be used to detect and quantify the differential expression of groups of genes.

In order to avoid excessive noise in the dataset we excluded all genes with very weak signals on the arrays (A ≤ 7; A = log2 [intensity in the Cy3 channel + intensity in the Cy5 channel]) from further analysis, resulting in the retention of data from 13 415 genes. The vast majority of BINs with differentially expressed genes contained genes that were both up- and down-regulated. Furthermore, after applying Benjamini-Hochberg correction for multiple comparisons most of the BINs did not, as a whole, show significant between-genotype differences in expression levels. Hence, most of the changes in gene expression were scattered amongst diverse biochemical pathways and functional groups. However, a few BINs did show significant differential expression, especially BINs related to the photosynthetic apparatus. Genes in these BINs were expressed more strongly in oePsbS than in both wild-type and *npq4 *plants (Table 2). Extremely few of the genes in these BINs appeared to be more strongly expressed in *npq4*. For instance, the only gene that was apparently up-regulated in *npq4 *plants in the "Photosynthetic light reactions" BIN encodes ELIP (early light-inducible protein); a protein known to be stress-induced and even employed as an abiotic stress marker [16]. Other BINs that were also significantly more strongly expressed in oePsbS plants contained plastidic ribosomal proteins and pentatricopeptide (PPR) repeat-containing proteins, which are known to be important for plastid gene regulation. The BINs "major CHO metabolism" and "minor CHO metabolism.raffinose family.raffinose synthases" tended to have higher expression levels in *npq4 *than in oePsbS plants, but the difference was of very weak significance (p ≈ 0.09), and there was no evidence of such a tendency *in npq4 *versus wild-type or oePsbS versus wild type comparisons.

**Table 2:** List of MAPMAN "BINs" showing evidence for differential expression

			**npq4 – wt**	**npq4 – oe**	**oe – wt**	**High exp in**
**BIN**	**name**	**elem**.	**p-value**	**p-value**	**p-value**	

1	PS	104	0.624	**0.000**	**0.000**	oePsbS

1.1	PS lightreaction	60	0.522	**0.000**	**0.000**	oePsbS

1.1.1	PS lightreaction photosystem II	26	0.205	**0.002**	0.381	

1.1.1.2	PS lightreaction photosystem II PSII polypeptide subunits	20	0.232	**0.019**	0.525	oePsbS

1.1.2	PS lightreaction photosystem I	13	0.982	0.019	**0.016**	oePsbS

1.1.2.2	PS lightreaction photosystem I PSIpolypeptide subunits	9	0.981	0.094	0.089	oePsbS

1.3	PS calvin cyle	27	0.819	**0.009**	**0.005**	oePsbS

2	major CHO metabolism	82	0.536	0.086	0.604	npq4

3.1.2	minor CHO metabolism raffinose family raffinose synthases	4	0.439	0.089	0.465	npq4

6	gluconeogenese/glyoxylate cycle	11	0.188	0.092	0.979	

16	secondary metabolism	266	0.065	0.272	0.992	

17.7	hormone metabolism jasmonate	18	0.053	0.053	0.631	npq4

17.7.1	hormone metabolism jasmonate synthesis-degradation	9	0.065	0.089	0.758	npq4

19	tetrapyrrole synthesis	26	0.188	0.066	0.840	oePsbS

26.19	misc plastocyanin-like	24	0.365	**0.012**	0.586	

29.2	protein synthesis	318	0.163	**0.001**	0.465	oePsbS

29.2.1	protein synthesis chloroplast/mito – plastid ribosomal protein	50	**0.050**	**0.030**	0.525	oePsbS

29.2.1.1	protein synthesis chloroplast/mito – plastid ribosomal protein plastid	24	0.229	**0.010**	0.378	oePsbS

29.5	protein degradation	1178	0.065	0.086	0.667	

34	Transport	646	0.557	**0.030**	0.301	npq4

34.13	transport peptides and oligopeptides	36	0.540	0.086	0.599	npq4

35.1.5	not assigned no ontology pentatricopeptide (PPR) repeat	322	0.188	**0.010**	0.254	oePsbS

List of MAPMAN "BINs" showing evidence for differential expression. Only "BINs" with p-values < 0.10, after Benjamini-Hochberg-correction, in *npq4 *vs. wild-type, *npq4 *vs. oePsbS (oe) or wild-type vs. oePsbS (oe) comparisons are listed. P-values < 0.05 in bold. Classes without informative annotations (like "Not assigned") are excluded. "High exp in" shows the direction of the change; oePsbS means that the BIN had higher expression in that genotype. Number of element (elem.) is number of genes in that category.

BINs involved in the metabolism of diverse secondary metabolites contained many genes with differential transcript abundance, but the patterns were not consistent within whole BINs. For example, many genes in the BIN "Anthocyanin metabolism" were up-regulated in *npq4*, in accordance with our previous finding that under many conditions *npq4 *mutants accumulate more anthocyanins than wild-type plants [5], but the BIN as a whole was not significantly up-regulated. Similarly, the expression levels of many genes involved in both the anabolism and catabolism of amino acids also differed significantly between genotypes, although there were no overall genotype-related trends in these BINs.

The differential expression of many genes with functions relating to plastid metabolism was also corroborated by a different analysis, in which we extracted expression data for all the genes corresponding to the 1590 nuclear-encoded genes with plastid-related functions on the cDNA macroarray, as performed by [17]. Analysis of the genes in this set (excluding those with A ≤ 7) showed that expression levels of genes representing seven of the 23 regulons, as identified by [17], differed significantly between genotypes (data not shown). For example, we obtained data for 60 of the 80 genes assigned to regulon 1 (which mainly consists of genes encoding photosystem subunits) and the expression levels of these genes were on average 35% higher in oePsbS than in *npq4 *plants.

Several genes in BINs related to redox regulation (e.g. the "Ascorbate/glutathione", "Glutaredoxin", "Peroredoxin" and "Dismutase/catalase" BINs) are involved in protection against photo-oxidative stress, and thus we expected their expression to be affected by changes in PsbS levels in field-grown plants. However, even though there were tendencies for some genes in the "Glutaredoxin" BIN to be up-regulated, and "Heme-oxidation proteins" to be down-regulated (data not shown), patterns of mRNA levels relating to genes in these BINs showed no overall differences between genotypes. The changes in PsbS levels may not have induced adjustments in the overall expression of genes encoding ROS protection systems, since the systems may have been sufficiently strong to cope with the oxidative stress experienced by plants of all three genotypes during the study period. However, these findings indicate that the main plant response to changes in PsbS expression, at least in their acclimation state at the time we sampled them, was not a localized increase in the capacity to cope with photo-oxidative stress, but rather a change in primary metabolism. Interestingly, many BINs relating to jasmonate (JA) synthesis were most strongly expressed in *npq4 *plants.

### The metabolic reprogramming may be mediated, at least in part, by jasmonate

How could changes in the level of PsbS have such profound effects on plant metabolism and transcription? Considering the function of PsbS as a key protein in the FDE regulation of light harvesting, one plausible signal transduction pathway could be through ROS signalling, but other mechanisms are certainly possible. This prompted us to compare the transcriptional response we observed in plants lacking PsbS with reported responses of *Arabidopsis *to various abiotic stresses. For this purpose, we extracted the 100 genes that were most significantly upregulated in *npq4 *relative to wild-type plants, according to B-statistics. Then, using Genevestigator , we compared these genes to sets of genes that have been found to be upregulated in published DNA micoarray analyses. Our list of induced genes had the largest overlap with the set of genes induced by ozone treatment; 20% of the genes on our list were upregulated more than 2-fold by ozone treatment and only 5% were downregulated more than 2-fold (Table 3). The sets of genes induced by many other treatments (e.g. several UV treatments, cold stress, low nitrate and heat stress) contained about equal numbers of genes up- and down-regulated in our dataset, indicating that the overall plant response to these stresses was not similar to that observed in *npq4*. After ozone, the most similar response was that reportedly provoked by mechanical wounding, for which 11% of the genes upregulated more than 2-fold, and none of the downregulated genes, were also present in our gene list. These findings indicate that PsbS depletion elicited a response that resembles plant responses to ozone or wounding.

**Table 3:** Overlap between microarray results

Treatment	Up-regulated	Down-regulated
*Abiotic stresses*		
Ozone	20	5
Oxidative	0	3
UV-A	1	1
UV-B	3	1
White light	3	2
Low nitrate	8	13
Cold	8	8
Drought	7	0
Heat	14	14
Wounding	11	0
		
*Hormones*		
IAA	2	3
GA3	3	1
Zeatin	2	2
ABA	10	2
Brassinolide	4	2
Salicylic Acid	4	10
Ethylene	4	4
Methyl jasmonate	17	4

The fraction (in %) of genes most significantly up- or downregulated in *npq4 *vs. wild-type *Arabidopsis *plants that show over-lap with different abiotic stresses and hormone treatments effects on gene expression. The data was compiled by comparing results from our study with other published studies using Genevestigator (see methods for details).

It also seemed plausible that such an effect on plant core metabolism could be mediated by plant hormone signalling. Therefore, we compared our gene list with sets of genes induced by various hormone treatments in the same way as above. Treatments with auxin, cytokinin, giberellin, ethylene or brassinolide do not apparently result in responses similar to that seen in *npq4*. The effect of abscissic acid showed some overlap (10% up, 2% down), whereas the response to salicylic acid was dissimilar (4% up, 10% down). However, plant responses to treatment with methyl jasmonate (MeJA) appear to be closest to those observed in field-grown plants lacking PsbS (17% up, 4% down), suggesting that at least part of the mechanism involved was a JA response.

**Figure 3:**
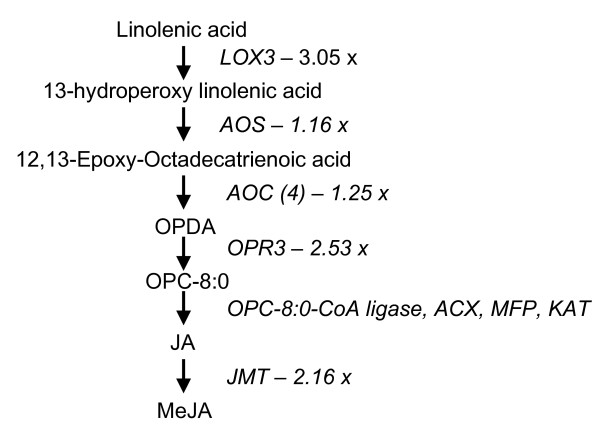
**Octadecanoid pathway gene induction in *npq4***. Induction of genes involved in the octadecanoid biosynthesis pathway in *npq4 *plants, as manifested by differences in levels of expression of jasmonate metabolism genes in rosette leaves of wild-type and *npq4 Arabidopsis *plants (measured by DNA microarrays). The numbers denote increases (fold changes) in levels of mRNAs encoding enzymes in the metabolic pathways in *npq4 *compared with wild-type plants.

Combining the transcriptomics data with the metabolomics data allows changes in the octadecanoid (JA/MeJA) pathway in plants with altered levels of PsbS to be clearly identified. JA biosynthesis is still only partly understood, but one of the first steps is known to be the release of linolenic acid (LA) from the chloroplast envelope [18]. Levels of linolenic acid were very similar to wild-type levels in *npq4 *plants, but they were higher in oePsbS plants (data not shown). However, mRNA levels for four of the enzymes – lipoxygenase (AtLOX3), allene oxide synthase (AOS), allene-oxide cyclase and jasmonate O-methyltransferase – involved in the conversion of linolenic acid to methyl jasmonate (Fig. 3) were up-regulated, as was the expression of hydroperoxide lyase, which converts 13(S)-hydroperoxylinolenic acid to 12-oxo-cis-9-dodecenoate. The metabolomics analysis provided no information on levels of metabolites downstream of linolenic acid. Some of them may correspond to as yet unidentified peaks in the chromatograms, while others may have been present in very low amounts or may not be detectable by our setup. For example, the end product of the pathway, methyl jasmonate, is a volatile that is unlikely to be detected with our analytical protocols.

**Figure 4:**
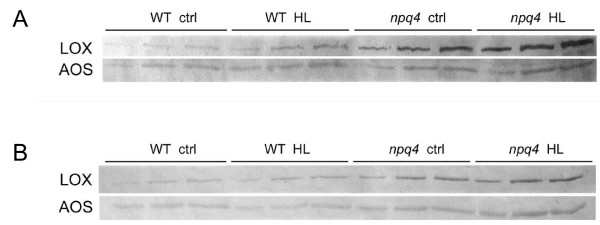
**Thylakoid protein profiles**. Immunoblotting of wild-type and *npq4 *protein preparations from plants grown under normal (control) light (PPFD = 100 μmol quanta m-2 s-1) and high light (HL, PPFD = 1600 μmol quanta m-2 s-1) with antibodies against LOX-C and AOS. (A) total leaf extracts loaded on basis of equal leaf area in three dilutions, from left to right 1X, 2X and 3X. (B) thylakoid membrane proteins loaded on basis of equal protein – determined by bicinchoninic acid assay (Pierce)-, from left to right 20, 40 and 60 microgram protein.

To confirm that responses seen at the transcriptional level were also present at the protein level the polypeptide composition of thylakoid membranes was analysed using 2D SDS-PAGE. Samples were analysed from wild-type and *npq4 *plants grown under both normal (NL) and high light (HL) conditions. Additional spots appeared to be present in gels displaying peptides from *npq4 *plants grown under HL, although complete analysis of the HL-induced polypeptide composition is beyond the scope of this study and will be presented elsewhere. However, of particular interest were two spots migrating to apparent Mw positions of 110 kDa and 106 kDa, both identified by mass spectroscopy as lipoxigenase (LOX-C), and a single spot, at approx 52 kDa, identified as AOS. The identification of these gene products as the major components of the spots in the 2D gels was confirmed by expressing the corresponding cDNAs in E. coli and preparing polyclonal antibodies to decorate the corresponding spots in immunoblots. An immunoblotting reaction on wild-type and *npq4 *whole leaf extracts showed the presence of LOX-C and AOS bands in *npq4 *samples even in NL conditions, while in the wild-type samples they were present in lower levels even when grown under HL (Fig. 4). In each genotype the intensity of the LOX-C and AOS bands was proportional to the level of stress experienced by the plant as determined by increases in zeaxanthin, tocopherol and lipid peroxides. An additional band with lower molecular weight (34 kDa), and not yet identified exhibited a similar behaviour in the Coomassie-stained SDS-gel. Immunoblotting was repeated after isolation of thylakoid membranes with essentially the same results. We conclude that LOX-C and AOS are found as thylakoid-associated proteins in the *npq4 *genotype and/or in HL conditions, and that their amount seems to be proportional to the level of light stress experienced by the plants (Fig. 4).

An SDS-PAGE gel displaying peptides from wild-type and *npq4 *thylakoids showed the presence of LOX-C and AOS bands in *npq4 *samples even in NL conditions, while in the wild-type samples they were present in lower levels even when grown under HL. In each genotype the intensity of the LOX-C and AOS bands, and that of a lower molecular weight band (34 kDa), was proportional to the level of stress experienced by the plant as determined by increases in zeaxanthin, tocopherol and lipid peroxides. We conclude that LOX-C and AOS are found as thylakoid-associated proteins in the *npq4 *genotype and/or in HL conditions, and that their amount seems to be proportional to the level of light stress experienced by the plants.

**Figure 5:**
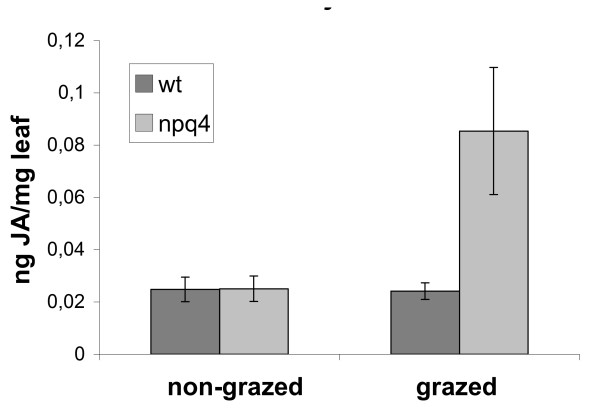
**Jasmonate levels in leaves of grazed and non-grazed field-grown *Arabidopsis *plants**. JA levels (in ng JA/mg leaf tissue) were quantified in individual plants of wild-type and *npq4 *genotypes, that has been grazed or not. Error bars denote ± SE.

Both our transcript and protein analyses indicated that JA signalling may, under field conditions, be increased in plants lacking PsbS. Therefore, to examine this possibility further, we measured the levels of JA in wild-type and *npq4 *plants grown in the field. When plants were not grazed we found no significant differences in JA levels between genotypes. However, when plants were subjected to moderate grazing JA levels were significantly elevated in *npq4 *plants, to levels that were on average more than three times higher than those seen in the wild-type (Fig 5). This suggests that JA signalling could be more readily induced in *npq4*.

### Increased JA signalling in *npq4 *plants may influence herbivore preferences

**Figure 6:**
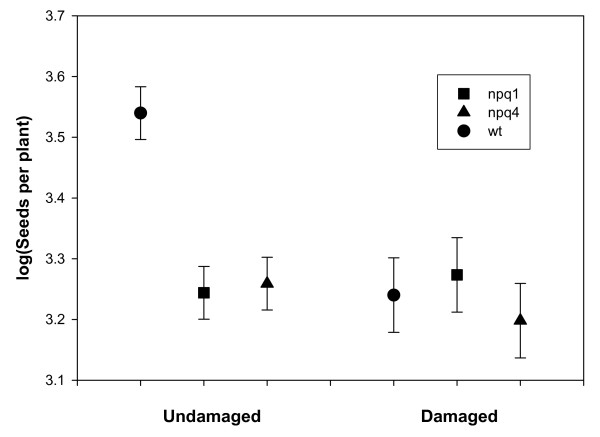
**Fitness after herbivory**. Seed production per plant by field-grown wild-type, *npq1 *and *npq4 Arabidopsis *plants. The three genotypes were grown in a randomized block design in the field, and two out of the six blocks were subject to grazing by naturally occurring herbivores. Seed production is shown as log10 value, error bars denote ± SE.

The finding that JA signalling could be more readily provoked in plants lacking PsbS provided a possible explanation for observations made during earlier field-studies on plants lacking PsbS or VDE (Violaxanthin de-epoxidase) [3]. These experiments were performed using a randomized block design over two years. In the first year, the relative performance of mutant and wild-type plants varied considerably among blocks, and the variation was associated with differences in grazing damage. In four of the six blocks there was little or no sign of grazing, and in these blocks the mutants lacking PsbS (and thus with reduced FDE capacity) showed a clear reduction in fitness compared with the wild-type plants. However, in the remaining two blocks all plants were considerably damaged by herbivores and there was no difference in fitness between the genotypes (Fig. 6). In the original study we left this between-block variation unexplained, since the overall fitness in both years was significantly reduced in the mutants compared with the wild-type. However, the results suggest that the herbivory negatively affected the fitness of wild-type plants more strongly than the fitness of the mutants.

**Figure 7:**
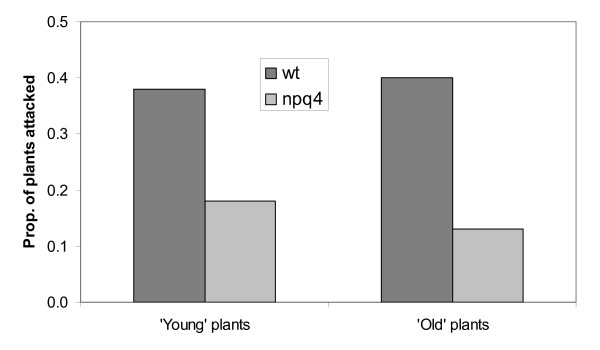
**Herbivory on wild-type and *npq4 *plants**. Levels of natural herbivory on *Arabidopsis *wild-type and *npq4 *plants scored in the common garden. 'Young' plants were introduced to the field after six days of growth, while 'old' plants were introduced to the field after 21 days of growth. Both number of plants attacked by herbivore and the proportion if leaf area eaten was scored.

Therefore, we performed a cafeteria experiment to determine herbivore preferences to test the hypothesis that these differences in herbivory were due to the differences in PsbS levels in the plants, and we also scored natural herbivory on plants growing in our experimental garden. A generalist herbivore (the netted slug; Deroceras reticulatum) preferred wild-type plants over plants lacking PsbS (One-tailed t-test, t = 1.604, df = 66, p = 0.057). Natural grazing, scored over the summer on plants of two different ages, was also higher on wild-type plants (Fig. 7). Overall, these findings could suggest that metabolic changes – either in primary or secondary metabolism – induced in plants lacking PsbS made the plants less palatable for herbivores and/or that more volatile repellents or less attractants are produced.

## Discussion

This study shows that under natural conditions *Arabidopsis *plants respond to changes in their FDE capacity quite dramatically. Our initial assumption was that a lack of PsbS, and hence FDE, would reduce the plants' Darwinian fitness as a consequence of changes in their photosynthetic performance. This may be true in part [5], but this study demonstrates that the plant's "core metabolism" (carbohydrate and amino acid metabolism) is also strongly affected, with large changes to the transcriptome occurring in plants lacking PsbS. Several amino acids (e.g. glutamine, glycine, aspartate and threonine) were highly depleted in plants lacking PsbS, whereas levels of others (e.g. leucine) were higher. Various carbohydrates also accumulated in plants lacking PsbS including (inter alia) galactose, raffinose and isomaltose, while sucrose levels were reduced. Knowledge of metabolic changes induced in *Arabidopsis *by different treatments is rather limited, but several of these compounds have been reported to accumulate under various kinds of stresses. For instance, there have been suggestions that the raffinose family oligosaccharides (RFOs) provide tolerance against drought, high salinity and cold stress [19]. Hence, the metabolic reprogramming that occurred in plants lacking PsbS could be described in general terms as a stress response. Since transcriptional changes induced in *Arabidopsis *by various treatments have been intensively examined in several large-scale DNA microarray projects, they are far better characterized than overall metabolome changes. This allowed us to compare our transcriptome data with responses induced by a variety of biotic and abiotic stresses, as well as hormone treatments.

Comparing microarray data obtained using different platforms is far from straightforward but there are examples showing that cross-platform comparisons can give reliable results. For example, a benchmarking of GST-based CATMA arrays (which we used), and Affymetrics arrays indicated that the results were largely consistent [20]. However, no study, including our own, has provided data on the degree of overlap between sets of transcripts in identical RNA samples identified as being upregulated in both CATMA and Affymetrics analyses. Hence, it is not easy to evaluate the absolute level of overlap between the set of genes induced in *npq4 *plants and those induced by other treatments, especially since our data were obtained under field conditions, while those presented in all other studies published to data were obtained from plants grown under controlled conditions in the lab. Nevertheless, the response seen in the *npq4 *genotype was most similar to those reportedly induced by ozone exposure or wounding, and also similar to a JA/MeJ response. The latter finding was further corroborated in four ways: comparison with other array data; detailed analysis of genes and transcripts associated with the JA/MeJa pathway in our plants; proteomic analysis; and measuring the JA levels within the plants. We hypothesize that a lack of PsbS in *Arabidopsis *plants leads to increased photooxidative stress and consequently induces a stress response. The signals involved are likely to be complex and to involve several hormones/signalling substances. However, since the response we found most closely resembled a JA/MeJA response in terms of the upregulated genes in microarray experiments, JA is probably one of the substances involved, and perhaps the most important. Additional studies using mutants with disturbances in different signalling pathways will hopefully provide further information regarding the nature of the signalling pathways involved.

It should be pointed out that plants probably have several mechanisms for sensing high light, and the signalling pathways involved in the responses observed in our study are unlikely to be identical to those that mediate general high light responses. For example, the effect of cryptochrome on high light-induced changes in gene expression [21] is apparently mediated by a different pathway from the response reported here, and the connection between ABA and high-light responses reported by [22] may involve different pathways too. However, it should be noted that there was some overlap between the genes induced in *npq4 *and those reportedly induced by ABA, although to a lower degree than the overlap seen with the JA response. Plants exposed to excessive light conditions need to modify many aspects of their growth and metabolism, since high light may be accompanied with any one or more of various other stressors, so multiple pathways could allow plants to respond to ecological challenges flexibly, with strong selective advantages.

In contrast to the abovementioned studies, here we compared genotypes that differed only in their capacity to dissipate excess light in the photosynthetic light-harvesting antenna, grown under identical environmental conditions. Thus, we focused on a single aspect of high-light signalling, mediated by signals directly generated at the photosynthetic reaction site. The results show that increased photooxidative stress at photosystem II is somehow sensed and a nuclear signal is generated that modifies gene expression. This in turn leads to a reprogramming of plant metabolism, with the implication that JA could be involved in the process. JA might even be the direct signal, and our finding that two enzymes involved in the JA biosynthesis pathway were enriched in the thylakoid membranes of PsbS-deficient plants may provide clues regarding the mechanism behind the response. Activation of the JA pathway is believed to occur at the site of JA biosynthesis; the chloroplastic membrane [23]. Therefore, JA is locally synthesized in response to light stress, and is also amplified by a positive feedback loop [24]. However, the level of JA was unaffected in *npq4 *plants unless they were provoked by herbivory. This does not exclude the direct involvement of the octadecanoid pathway in the response; increases could be induced in the flux through the pathway or in levels of either MeJA or JA-Ile, which may be the active species [25]. However, it is also possible that plants lacking PsbS could be more "primed" and respond more actively to herbivory by increasing JA levels. At this point it is not possible to distinguish between these possibilities, but we hope to address these possibilities in future studies.

Regardless of the exact role that JA plays in the process, the metabolic reprogramming may be sufficient to explain the lower fitness observed in plants lacking PsbS, since JA-induced responses are known to reduce seed production [26]. Furthermore, *Arabidopsis *plants treated with JA have an appearance that, in our experience, closely resembles that of plants exposed to moderate light stress, with anthocyanin accumulation, changes in leaf morphology and growth retardation (unpublished results). Overall, these findings suggest that plants lacking PsbS allocate resources away from reproduction towards defence. It has become increasingly apparent that stress responses are very complex. For instance, different herbivores may elicit specific responses in attacked plants and jasmonate signalling may affect different herbivores in different ways [27]. Furthermore, although responses to various abiotic stresses share several common features, they also exhibit many highly specific features. Since most stress signalling has been shown to involve ROS in some way, one speculative possibility is that the changes we have observed could correspond to a more basal metabolic shift from reproduction and/or growth towards defence that increases the plant's ability to respond competently to stimuli elicited by specific stresses.

PsbS levels in wild-type plants do not appear to be "saturated", suggesting that selection acts against the production of higher levels of PsbS, even though it would increase the FDE capacity and hence potentially provide more photoprotection. Although this must theoretically hold true for the expression levels of all genes, it is not easy to demonstrate experimentally. An interesting implication of our data is that too much photoprotection could compromise "stress signalling", either in a more general sense, as discussed above, or more specifically through the involvement of JA/MeJA. Hence, plants with elevated PsbS levels could have less competence to cope with other stresses, and recent data indicate that this is indeed the case (Frenkel et al, in preparation). Overall, these findings suggest that there might be a tradeoff between photoprotection and tolerance to other stresses, as indicated by our data showing differences in herbivore preferences between wild-type plants and plants lacking PsbS.

Many mutants show marked phenotypic deviations from corresponding wild types, but mutations in most genes lead to more subtle changes in knock-out or antisense plants, with the lack of observable phenotypic deviations being the rule rather than the exception (see e.g. [28,29]). Functional redundancy, of course, could be one reason for this, but it is clear that in many cases no observable phenotypic change may occur because the plants are not grown under suitable conditions for such changes to appear. Plant metabolic networks have highly complex architectures and the ability to compensate for enzyme deficiencies, or even an insufficiency in an entire pathway, using alternative pathways or substances that can perform similar functions, is likely to be of great adaptive value. Growing plants under naturally stressful conditions, rather than under controlled conditions in the lab, inevitably generates data that are complex and difficult to interpret. To our knowledge, the present study provides the first demonstration that metabolomic and transcriptomic analyses of mutants and over-expressers grown in the field can provide new and unexpected conclusions about individual gene products. We have been able to separate the genotypic differences from uncontrolled variation, and metabolomic analyses performed in the field and the laboratory clearly demonstrated that genotypic differences in expression patterns observed in field-grown material were not necessarily present under controlled conditions. It should be stressed that the responses described here were not detected under standard growth conditions in the lab. Therefore, we believe that studies like this, using plants grown under natural conditions, are essential for appreciating the full complexity of plants' genetic and metabolic composition and their interactions with the environment.

## Conclusion

We have performed metabolomic, transcriptomic, proteomic, hormone and herbivore preference analyses of plants containing differing amounts of PsbS, both in the lab and the field. The results show that the level of PsbS is a determinant of plant fitness, metabolism and gene expression, perhaps through influencing the JA response.

## Methods

### Plant material and growth conditions

The plants used in the experiments represented three genotypes: *Arabidopsis thaliana *cv *Columbia-0 *(referred to as wild-type, wt), oePsbS [30] and *npq4*-1 [1]. The *npq4-1 *mutant has a fast-neutron induced deletion of the gene encoding PsbS. Plants with the *npq4-1 *genotype had been backcrossed for three generations after the mutagen treatment. Plants overexpressing the PsbS gene were generated by *Agrobacterium tumefaciens*-mediated transformation of *Arabidopsis thaliana *(*Col-0*) plants, using the plasmid pXPL7 containing a 3198-bp fragment of the BAC clone F9J23. Plants of the line used here (designated line 5) were backcrossed for one generation after homozygous lines were identified (F3 generation).

For the fitness experiment, 40 plants representing each of the three genotypes were grown as described in [3]. The plants were grown in an experimental garden under natural conditions; they received no fertilization and were watered ad lib when dry. Since oePsbS is a transgenic line we abided by the terms of our growing permit, which stipulates that all transgenic *Arabidopsis *plants grown in the field must be covered by a net from the time the first plant bolts. This ensures insects are excluded from the site and thus prevents accidental insect-mediated cross-pollination with wild plants. After flowering had ceased all the siliques were counted, and seeds were also counted in three randomly selected siliques from each plant. The mean number of seeds per silique was then multiplied by the number of siliques produced per plant to estimate total seed production.

For the metabolomic and transcriptomic measurements, the three genotypes (wild-type, *npq4 *and oePsbS) were pre-grown in a growth chamber under short day conditions (8-hour light periods with 400 μmol quanta m-2 s-1) with a 23°C/18°C temperature regime and relative humidity of 70%. All plants were moved to an experimental garden after five weeks of growth and then left there for a further five days. At the field site the photon flux density (PPFD) ranged from very low levels up to 600 W/m2 (ca. 2300 μmol quanta m-2 s-1) during the (ca. 15-hour) photoperiods and was highly variable. The temperature varied between 16° and 28°C, and the relative humidity (RH) between 30 and 100%. At 11 am on the fifth day, one leaf of each of 10 plants representing each genotype was harvested for metabolite measurements, and two pools of 3–4 leaf rosettes from each genotype were harvested for transcript analysis. Samples were frozen immediately after harvesting and stored at -80°C until further use.

For the metabolomic measurements from the constant conditions, the three genotypes were grown in a growth chamber under short day conditions (8-hour light periods with 200 μmol quanta m-2 s-1) at ca 23°C temperature and relative humidity of about 30%.

### Metabolite measurements

Approximately 10 mg of each sample was extracted and analyzed for metabolite profiling, according to [7] with some minor modifications. To the chloroform:MeOH:H2O (6:2:2) extraction mixture (1 ml) used for each sample, the following stable isotope reference compounds were added: [2H7]-cholesterol, [13C3]-myristic acid, [13C4]-hexadecanoic acid, [2H4]-succinic acid, [13C5, 15N]-glutamic acid, [13C5]-proline, [13C4]-disodium α-ketoglutarate, [13C12]-sucrose, [2H4]-putrescine, [2H6]-salicylic acid and [13C6]-glucose. The extraction was performed using an MM 301 Vibration Mill (Retsch GmbH & Co. KG, Haan, Germany) at a frequency of 30 Hz s-1 for 3 min after adding 3 mm tungsten carbide beads (Retsch GmbH & Co. KG, Haan, Germany) to each tube to increase the extraction efficiency. The extraction was followed by centrifugation in an Eppendorf centrifuge (Model 5417C) for 10 min at 14 000 rpm, after which 200 μl of the supernatant was transferred to a GC-vial and evaporated to dryness. The samples were then derivatized using 30 μL of methoxyamine hydrochloride (15 mg mL-1) in pyridine for 1 h at 70°C, followed by 16 h at room temperature, and subsequently trimethylsilylated by adding 30 μL of MSTFA with 1% TMCS and incubating for 1 h at room temperature. After silylation, 30 μL of heptane containing 45 ng μl-1 methyl octadecanoate was added. The samples were analyzed, together with blank control samples and a series of n-alkanes, according to [7], by GC/TOFMS (C12-C40), to facilitate calculation of retention indices [11].

One μL of each derivatized sample was injected splitless by an Agilent 7683 autosampler (Agilent, Atlanta, GA, USA) into an Agilent 6890 gas chromatograph equipped with a 10 m, 0.18 mm i.d. fused silica capillary column with a chemically bonded 0.18 μm DB 5-MS stationary phase (J&W Scientific, Folsom, CA, USA). The injector temperature was 270°C, the septum purge flow rate was 20 ml min-1 and the purge was turned on after 60 s. The gas flow rate through the column was 1 ml min-1, the column temperature was held at 70°C for 2 min, then increased by 40°C min-1 to 320°C and held there for 2 min. The column effluent was introduced into the ion source of a Pegasus III time-of-flight mass spectrometer (Leco Corp, St Joseph, MI, USA). The transfer line and the ion source temperatures were 250°C and 200°C, respectively. Ions were generated by a 70 eV electron beam at an ionization current of 2.0 mA, and 30 spectra s-1 were recorded in the mass range 50 to 800 m/z. The acceleration voltage was turned on after a solvent delay of 170 s. The detector voltage was 1660 V.

All non-processed MS-files from the metabolic analysis were exported from the ChromaTOF software in NetCDF format into MATLAB™ 6.5 software (Mathworks, Natick, MA, USA), where all data pre-treatment procedures, such as base-line correction, chromatogram alignment, data compression and Hierarchical Multivariate Curve Resolution (H-MCR) were performed using custom scripts according to [8]. All manual integration was performed using ChromaTOF 2.00 software (Leco Corp, St Joseph, MI, USA) and all multivariate statistical investigations (PCA, PLS-DA) were performed using Simca software 10.5.0.0 (Umetrics, Umeå, Sweden). The following statistics for the PLS-DA models are discussed at various points throughout this paper: R2X is the cumulative modelled variation in X, R2Y is the cumulative modelled variation in Y, and Q2Y is the cumulative predicted variation in Y, according to cross-validation. The range of these parameters is 0–1, where 1 indicates a perfect model.

Significantly differing metabolites were detected by interpreting the first weight vector (w1), as described by [9], from the *npq4*/oePsbS PLS-DA model, together with the 95% confidence intervals calculated using jack-knifing [10].

### Transcript measurements

**Figure 8:**
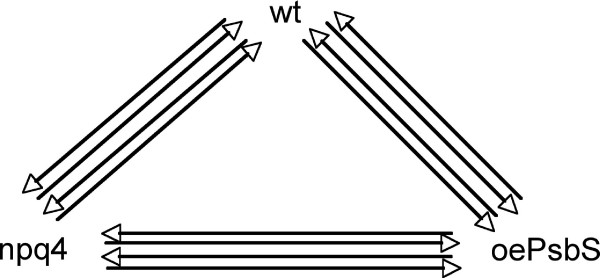
**Microarray experimental design**. Hybridizations performed with samples from the tree genotypes. Two biological replicates were analysed per genotype and arrows illustrates each hybridization (four per biological replicate, two labelled with Cy3 and two with Cy3, and two against each of the other genotypes), in total twelve hybridizations.

The samples for transcriptomic analysis were taken from the same plants at the same time (i.e. five days after transfer to the field) as the samples for the metabolomic analysis to maximize consistency and facilitate comparative analyses. Like the metabolome, the plant transcriptome in the field is likely to be highly influenced by factors that are difficult to control, making it necessary to analyze several biological replicates. However, the sample throughput rates are lower for microarrays than for metabolomic analyses so we adopted a different sampling strategy to maximize the reliability of the dataset. Each sample for microarray analysis contained a pool of whole rosettes (minus the leaf taken for the metabolomic analysis) from three or four plants of approximately equal size. In order to estimate whether this sampling strategy was capable of sufficiently reducing noise from non-relevant biological variation, we prepared two independent pooled samples that were included as biological replicates, and each biological replicate was analyzed in two technical replicates using a looped experimental design (Fig 8). Samples were ground in a mortar in liquid nitrogen and RNA was extracted using an RNeasy Plant Mini Kit (Qiagen, Science, Maryland, USA). The extracted total-RNA was amplified using a Message Amp™ II aRNA kit (Ambion, Austin, Texas, USA), and the amplified RNA was then reverse-transcribed with a custom aa-dUTP mix (Sigma-Aldrich, Stockholm, Sweden) and N9 random primers (Invitrogen AB, Stockholm, Sweden) using Superscript II (Invitrogen AB, Stockholm, Sweden) for indirect labelling. cDNA was cleaned up using Microcon YM-30 columns (Millipore AB, Solna, Sweden). The cDNAs were coupled to Cy3 and Cy5 dyes (Amersham Biosciences, Uppsala, Sweden) and cleaned up using a CyScribe GFX purification kit (Amersham Biosciences, Uppsala, Sweden). Hybridizations were done on CATMA cDNA microarrays, containing 25 392 spots [31], using an Automated Slide Processor (Amersham Biosciences, Umeå, Sweden). The slides were scanned at 10 μm resolution, using a ScanArray Light scanner (PerkinElmer Sverige AB, Upplands Väsby, Sweden) with ScanArray Express 2.1 at three different laser intensities; 75, 80 and 100% with the photomultiplier tube (PMT) constant at 75%.

### Data analysis

Raw data were extracted from the Tiff images generated by ScanArray Express 2.1 using GenePix Pro 5.1 software (Axon Instruments, CA, USA). The threshold settings for the spot diameter resize feature were < 75% and > 150%, and the composite pixel intensity (CPI) was set to 300. The data were uploaded and normalized, filtered and analyzed within UPSC-BASE [32]. The complete set of data can be found at , experiment UMA-0060. In UPSC-BASE, UmeaSAMED restricted linear scaling (RLS) was performed to combine the different intensity scan data, thereby increasing the linear range of the signals . B-statistics were used to describe the quality of the technical and biological replicates. Two biological and two technical replicates were used and a total of 12 slides were hybridized with probes from the three genotypes (Fig 8). Before averaging the data for the biological replicates multivariate statistical investigations (PCA) were performed using Simca 10.5 software (Umetrics, Umeå, Sweden). For analyses involving MAPMAN software, datasets were exported from UPSC-BASE with a MAPMAN/AraCyc export tool. Multiple testing corrections were done in MAPMAN using the Wilcoxon Rank Sum Test and Benjamini Hochberg correction.

### Protein analysis

Thylakoid membranes were purified from wild-type and *npq4 *leaves grown at different light and temperature conditions as previously described [33]. 1D and 2D SDS-PAGE was performed using a Tris-sulphate buffer system [34] in a 12–18% acrylamide gradient. Gels were stained with Coomassie R. Spots were excised, vacuum-dried and subjected to mass spectroscopy after trypsin digestion according to standard protocols. cDNAs for LOX-C and AOS were obtained from the Nottingham Arabidopsis Centre and expressed in E. coli using a pQE-50 vector including a 6-HIS tag at the N-terminus. The recombinant proteins were isolated using a Ni++ column and further purified by SDS-PAGE and electro-elution of Coomassie-stained bands. Purified proteins were injected into rabbits and antisera were obtained. Immunoblotting was performed following 1D or 2D SDS-PAGE using alkaline phosphatase-coupled antibodies for detection. Zeaxanthin and tocopherol contents were determined by HPLC analysis of acetone extracts, and levels of peroxidised lipids were determined by measuring malonyl-dialdehyde (MDA) derivatives, separated and quantified by HPLC according to methods described by [35].

### Herbivory

We performed a cafeteria experiment to test for differences in herbivore preferences for the genotypes, as follows. We collected netted slugs (*Deroceras reticulatum*, Mullusca, Gastropoda) from the experimental site. Plants for the cafeteria experiments (pair-wise comparisons between the genotypes) were sown in 5 cm wide pots and then vernalised for 4–5 days at 4°C. After the vernalisation they were transferred to a growth chamber with short day conditions (8-hour light periods with 150 μmol quanta m-2 s-1), a 23°C/18°C temperature regime and constant relative humidity of 70% for ca. six weeks. After reaching a rosette size large enough to punch out several leaf discs of 1 cm diameter, they were brought to the experimental field site and exposed to natural, varying light conditions. Plants were taken for the experiments after 4–7 days in the field.

Eight leaf discs (1 cm diameter) from each of the two genotypes were taken and placed on wet filter paper in a 14.5 cm Petri dish, arranged in chess-board pattern, prior to introducing an individual of Deroceras. Pictures of the Petri dishes were taken after 15 hours, and the leaf area eaten during the experiment was quantified by image analysis using Scion Image . Every cafeteria experiment consisted of 10 replicates in paired combinations of the genotypes, and repeated on 7 different days giving a total of 70 runs.

During the summer of 2007, we scored naturally occurring herbivory in our experimental garden. To examine whether age (and size) of the plant affected herbivory levels, we used plants that were both 6 and 21 days old. The plants that were 21 days old were transferred to the field seven days after the younger plants. All plants were scored for leaf damage four times during the summer (after 18, 25, 29 and 34 days in the field according to the ages of the younger plants). For the analysis, we defined plants that had lost at least one leaf or more as damaged by herbivores.

## Authors' contributions

MF carried out the controlled condition metabolomics experiment, analysed the microarray data and carried out the herbivory analysis (except the initial dataset), CK carried out oePsbS fitness experiments, the metaboloics/transciptomcs field experiment including sampling and the initial analysis of microarray data, HJJ carried out the JA analysis and participated in the herbivore scoring, OS carried out the microarray analysis and LDO the proteomics analysis, JÅ analysed the initial herbivore damage dataset, RB supervised the proteomics analysis, TM carried out the metabolomics analysis and supervised the JA analysis, JM supervised the herbivore experiments, experimental design and statistical analysis SJ coordinated the study. MF, CK, RB and TM drafted various parts of the ms, SJ, together with JM, assembled the ms.

## Supplementary Material

Additional file 1 **GC/TOFMS peaks according to PLS-DA identified as important for explaining the difference between genotypes.** GC/TOFMS peaks according to PLS-DA identified as important for explaining the difference between genotypes. ^a^Peaks are named according to UPSC- in-house mass spectra library. ^b^Annotation of peaks were performed by comparing mass spectrum and retention index (RI) with the ^c^UPSC in-house mass spectra library or the or the mass spectra library maintained by the Max Planck Institute (MPI) in Golm . ^d^Peaks annotated or classified according "M000000..." are identical or similar to non-annotated mass spectra in the MPI-library. Naming refers to MPI-spectra numbering. ^e^Tentatively identified. ^f^First loading vector (w[1]) from the PLS-DA model between *npq4 *and oePsbS plants describes the importance of different GC/MS peaks for explaining the differences between the two genotypes; The values can vary between -1 and +1, and negative values are peaks correlated with oePsbS, positive values are peaks correlated with *npq4*. UPSC mass spectra will be available for download on UPSC homepage. Click here for file

Additional file 2 **Complete DNA microarray dataset.** Excel spreadsheet showing complete DNA microarray dataset. Click here for file

Additional file 3 **The 23 genes with the highest probability for differential gene expression between *npq4 *and oePsbS.** Positive p-values correspond to higher expression in oePsbS, and negative values to higher expression in *npq4*. Click here for file
