# Supplementary material for: Improper excess light energy dissipation in Arabidopsis results in a metabolic reprogramming
Source: BMC Plant Biol. 2009 Jan 26;9:12. doi: 10.1186/1471-2229-9-12 (PMC2656510; doi:10.1186/1471-2229-9-12)
Supplement: Additional file 1 — GC/TOFMS peaks according to PLS-DA identified as important for explaining the difference between genotypes. GC/TOFMS peaks according to PLS-DA identified as important for explaining the difference between genotypes. aPeaks are named according to UPSC- in-house mass spectra library. bAnnotation of peaks were performed by comparing mass spectrum and retention index (RI) with the cUPSC in-house mass spectra library or the or the mass spectra library maintained by the Max Planck Institute (MPI) in Golm . dPeaks annotated or classified according "M000000..." are identical or similar to non-annotated mass spectra in the MPI-library. Naming refers to MPI-spectra numbering. eTentatively identified. fFirst loading vector (w[1]) from the PLS-DA model between npq4 and oePsbS plants describes the importance of different GC/MS peaks for explaining the differences between the two genotypes; The values can vary between -1 and +1, and negative values are peaks correlated with oePsbS, positive values are peaks correlated with npq4. UPSC mass spectra will be available for download on UPSC homepage. [file 1471-2229-9-12-S1.doc]

**Supplementary Table 1.** GC/TOFMS peaks according to PLS-DA identified as important for explaining the difference between genotypes. ^a^Peaks are named according to UPSC- in-house mass spectra library. ^b^Annotation of peaks were performed by comparing mass spectrum and retention index (RI) with the ^c^UPSC in-house mass spectra library or the or the mass spectra library maintained by the Max Planck Institute (MPI) in Golm (http://csbdb.mpimp-golm.mpg.de/csbdb/gmd/gmd.html). ^d^Peaks annotated or classified according “M000000…” are identical or similar to non-annotated mass spectra in the MPI-library. Naming refers to MPI-spectra numbering. ^e^Tentatively identified. ^f^First loading vector (w[1]) from the PLS-DA model between npq4 and oePsbS plants describes the importance of different GC/MS peaks for explaining the differences between the two genotypes; The values can vary between -1 and +1, and negative values are peaks correlated with *oePsbS*, positive values are peaks correlated with *npq4*.

UPSC mass spectra will be available for download on UPSC homepage.

| **UPSC mass spectra library number^a^** | **Annotation^b^** | **Classification** | **RI** | **Library^c^** | **First loading vector (w1)^f^** |
| --- | --- | --- | --- | --- | --- |
| UPSC_10313_GCTOF_Ath _RI_1141 | Unknown |  | 1141 |  | -0,0880419 |
| UPSC_10314_GCTOF_Ath _RI_1185 | Unknown |  | 1185 |  | -0,0560055 |
| UPSC_10315_GCTOF_Ath _RI_1255 | Unknown |  | 1255 |  | -0,060942 |
| UPSC_10316_GCTOF_Ath _RI_1258 | Unknown |  | 1258 |  | -0,0880275 |
| UPSC_10317_GCTOF_Ath _RI_1268 | Ethanolamine 3TMS |  | 1268 | UPSC | -0,070821 |
| UPSC_10318_GCTOF_Ath _RI_1275 | Leucine 2TMS |  | 1275 | UPSC | 0,0653465 |
| UPSC_10319_GCTOF_Ath _RI_1276 | Unknown |  | 1276 |  | -0,0803575 |
| UPSC_10320_GCTOF_Ath _RI_1278 | Glycerol 3TMS |  | 1278 | UPSC | -0,0759837 |
| UPSC_10321_GCTOF_Ath _RI_1304 | Glycine 3TMS |  | 1304 | UPSC | -0,0937442 |
| UPSC_10322_GCTOF_Ath _RI_1318 | Succinic acid 2TMS |  | 1318 | UPSC | 0,0615862 |
| UPSC_10323_GCTOF_Ath _RI_1353 | Fumaric acid 2TMS |  | 1353 | UPSC | -0,0749069 |
| UPSC_10324_GCTOF_Ath _RI_1364 | Serine 3TMS |  | 1364 | UPSC | -0,0853169 |
| UPSC_10325_GCTOF_Ath _RI_1372 | Unknown |  | 1372 |  | -0,0821176 |
| UPSC_10326_GCTOF_Ath _RI_1385 | Threonine 3TMS |  | 1385 | UPSC | -0,0872048 |
| UPSC_10327_GCTOF_Ath _RI_1426 | β-Alanine 3TMS |  | 1426 | UPSC | -0,0655911 |
| UPSC_10328_GCTOF_Ath _RI_1436 | Unknown |  | 1436 |  | -0,0722798 |
| UPSC_10329_GCTOF_Ath _RI_1447 | Unknown |  | 1447 |  | -0,0716972 |
| UPSC_10330_GCTOF_Ath _RI_1454 | Piperidin-2-one, 3-amino 2TMS^e^ |  | 1454 | MPI | -0,0931019 |
| UPSC_10331_GCTOF_Ath _RI_1455 | Unknown |  | 1455 |  | -0,0836642 |
| UPSC_10332_GCTOF_Ath _RI_1467 | Citramalic acid 3TMS |  | 1467 | UPSC | -0,0634817 |
| UPSC_10333_GCTOF_Ath _RI_1485 | Malic acid 3TMS |  | 1485 | UPSC | -0,0679729 |
| UPSC_10334_GCTOF_Ath _RI_1517 | Aspartate 3TMS |  | 1517 | UPSC | -0,0764279 |
| UPSC_10335_GCTOF_Ath _RI_1521 | Pyroglutamic acid 2TMS |  | 1521 | UPSC | -0,0805217 |
| UPSC_10336_GCTOF_Ath _RI_1526 | 4-Aminobutyric acid 3TMS (GABA) |  | 1526 | UPSC | 0,0599035 |
| UPSC_10337_GCTOF_Ath _RI_1555 | Threonic acid 4TMS |  | 1555 | UPSC | 0,0655935 |
| UPSC_10338_GCTOF_Ath _RI_1556 | Unknown |  | 1556 |  | 0,0692402 |
| UPSC_10339_GCTOF_Ath _RI_1568 | Unknown |  | 1568 |  | -0,0818228 |
| UPSC_10340_GCTOF_Ath _RI_1577 | M000000_A159003-101_MST_1585.2_EITTMS^d^ |  | 1577 | MPI | 0,0833047 |
| UPSC_10341_GCTOF_Ath _RI_1584 | Unknown | Pentose | 1584 |  | -0,0681406 |
| UPSC_10342_GCTOF_Ath _RI_1611 | Unknown |  | 1611 |  | -0,0757758 |
| UPSC_10343_GCTOF_Ath _RI_1624 | Unknown |  | 1624 |  | -0,0581381 |
| UPSC_10344_GCTOF_Ath _RI_1641 | Unknown |  | 1641 |  | 0,0660911 |
| UPSC_10345_GCTOF_Ath _RI_1649 | Xylose MeOX 4TMS_2 |  | 1649 | UPSC | 0,0571818 |
| UPSC_10346_GCTOF_Ath _RI_1669 | Ribose MeOX 4TMS |  | 1669 | UPSC | 0,0922329 |
| UPSC_10347_GCTOF_Ath _RI_1685 | Unknown |  | 1685 |  | 0,0683982 |
| UPSC_10348_GCTOF_Ath _RI_1696 | Glucose, 1,6-anhydro, beta-D 3TMS^e^ |  | 1696 | MPI | 0,0565485 |
| UPSC_10349_GCTOF_Ath _RI_1711 | Unknown |  | 1711 |  | 0,071718 |
| UPSC_10350_GCTOF_Ath _RI_1717 | Arabitol 5TMS |  | 1717 | UPSC | 0,0645607 |
| UPSC_10351_GCTOF_Ath _RI_1731 | Unknown |  | 1731 |  | -0,0920788 |
| UPSC_103529_GCTOF_Ath _RI_1733 | M000000_A174001-101_MST_1744.9_EITTMS^d^ |  | 1733 | MPI | 0,076868 |
| UPSC_10353_GCTOF_Ath _RI_1754 | Unknown | M000000_A180004-101_MST_1795.4_EITTMS^d^ | 1754 | MPI | 0,0711219 |
| UPSC_10354_GCTOF_Ath _RI_1755 | Glycerol-3-phosphate 4TMS |  | 1755 | UPSC | -0,0696309 |
| UPSC_10355_GCTOF_Ath _RI_1762 | M000000_A177004-101_MST_1770.9_EITTMS^d^ |  | 1762 | MPI | 0,0849697 |
| UPSC_10356_GCTOF_Ath _RI_1769 | Glutamine 3TMS |  | 1769 | UPSC | -0,0855137 |
| UPSC_10357_GCTOF_Ath _RI_1770 | Unknown | Hexose | 1770 |  | 0,0737837 |
| UPSC_10358_GCTOF_Ath _RI_1783 | M000000_A180004-101_MST_1795.4_EITTMS^d^ |  | 1783 | MPI | 0,0651101 |
| UPSC_10359_GCTOF_Ath _RI_1814 | Citric acid 4TMS |  | 1814 | UPSC | -0,0619842 |
| UPSC_10360_GCTOF_Ath _RI_1833 | Unknown |  | 1833 |  | 0,0650169 |
| UPSC_10361_GCTOF_Ath _RI_1835 | Unknown |  | 1835 |  | 0,0605711 |
| UPSC_10362_GCTOF_Ath _RI_1837 |  | Unsaturated carbon chain | 1837 |  | -0,0625181 |
| UPSC_10363_GCTOF_Ath _RI_1839 | Dehydroascorbic acid dimer TMS^e^ |  | 1839 | MPI | 0,0715501 |
| UPSC_10364_GCTOF_Ath _RI_1843 | Unknown |  | 1843 |  | 0,0739107 |
| UPSC_10365_GCTOF_Ath _RI_1865 | Sorbose MeOX 5TMS_1 |  | 1865 | UPSC | 0,091719 |
| UPSC_10366_GCTOF_Ath _RI_1865 | Fructose MeOX 5TMS_1 |  | 1865 | UPSC | 0,0830273 |
| UPSC_10367_GCTOF_Ath _RI_1874 | Fructose MeOX 5TMS_2 |  | 1874 | UPSC | 0,0919417 |
| UPSC_10368_GCTOF_Ath _RI_1883 | Unknown |  | 1883 |  | -0,0881487 |
| UPSC_10369_GCTOF_Ath _RI_1891 | Glucose MeOX 5TMS_1 |  | 1891 | UPSC | 0,0914832 |
| UPSC_10370_GCTOF_Ath _RI_1899 | Unknown |  | 1899 |  | 0,0670038 |
| UPSC_10371_GCTOF_Ath _RI_1909 | Glucose MeOX 5TMS_2 |  | 1909 | UPSC | 0,0911651 |
| UPSC_10372_GCTOF_Ath _RI_1933 | Tyrosine 3TMS |  | 1933 | UPSC | 0,0567777 |
| UPSC_10373_GCTOF_Ath _RI_1940 | Unknown |  | 1940 |  | 0,0606517 |
| UPSC_10374_GCTOF_Ath _RI_1969 | Unknown | M000000_A196004-101_MST_1953.9_EITTMS^d^ | 1969 | MPI | 0,0775465 |
| UPSC_10375_GCTOF_Ath _RI_1980 | Unknown |  | 1980 |  | 0,0844107 |
| UPSC_10376_GCTOF_Ath _RI_1986 | Gluconic acid 6TMS |  | 1986 | UPSC | 0,0776876 |
| UPSC_10377_GCTOF_Ath _RI_2001 | Unknown |  | 2001 |  | 0,0679324 |
| UPSC_10378_GCTOF_Ath _RI_2081 | myo-Inositol 6TMS |  | 2081 | UPSC | 0,0785395 |
| UPSC_10379_GCTOF_Ath _RI_2098 | M000000_A211001-101_MST_2105.7_EITTMS^d^ |  | 2098 | MPI | 0,071253 |
| UPSC_10380_GCTOF_Ath _RI_2207 | Linoleic acid TMS |  | 2207 | UPSC | -0,0935336 |
| UPSC_10381_GCTOF_Ath _RI_2214 | alpha-Linolenic acid TMS |  | 2214 | UPSC | -0,0705547 |
| UPSC_10382_GCTOF_Ath _RI_2240 | Stearic acid TMS |  | 2240 | UPSC | -0,085515 |
| UPSC_10383_GCTOF_Ath _RI_2246 | Spermidine 5TMS |  | 2246 | UPSC | -0,0858709 |
| UPSC_10384_GCTOF_Ath _RI_2303 | Monogalactosyl glycerol 6TMS^e^ |  | 2303 | MPI | 0,0771204 |
| UPSC_10385_GCTOF_Ath _RI_2348 | Unknown | M000000_A237002-101_MST_2370.2_EITTMS^d^ | 2348 | MPI | 0,0710829 |
| UPSC_10386_GCTOF_Ath _RI_2389 | Unknown |  | 2389 |  | 0,0606903 |
| UPSC_10387_GCTOF_Ath _RI_2395 | Unknown |  | 2395 |  | 0,0603765 |
| UPSC_10388_GCTOF_Ath _RI_2395 | myo-Inositol-1-phosphate 7TMS |  | 2395 | UPSC | 0,047486 |
| UPSC_10389_GCTOF_Ath _RI_2437 | Unknown | Fatty ester | 2437 |  | -0,0791421 |
| UPSC_10390_GCTOF_Ath _RI_2489 | M000000_A250001-101_MST_2495.5_EITTMS^d^ |  | 2489 | MPI | 0,0706813 |
| UPSC_10391_GCTOF_Ath _RI_2497 | M000000_A251003-101_MST_2507.9_EITTMS^d^ |  | 2497 | MPI | 0,06327 |
| UPSC_10392_GCTOF_Ath _RI_2602 | Unknown |  | 2602 |  | 0,0655945 |
| UPSC_10393_GCTOF_Ath _RI_2624 | Sucrose 8TMS |  | 2624 | UPSC | -0,0601188 |
| UPSC_10394_GCTOF_Ath _RI_2751 | Maltose 8TMS |  | 2751 | UPSC | 0,0590419 |
| UPSC_10395_GCTOF_Ath _RI_2833 | Unknown | Fatty ester | 2833 |  | -0,0590211 |
| UPSC_10396_GCTOF_Ath _RI_2852 | Isomaltose 8TMS |  | 2852 | UPSC | 0,0648014 |
| UPSC_10397_GCTOF_Ath _RI_2857 | Unknown | Sterol | 2857 |  | -0,0580643 |
| UPSC_10398_GCTOF_Ath _RI_2915 | Unknown |  | 2915 |  | 0,0446718 |
| UPSC_10399_GCTOF_Ath _RI_2959 | Galactinol 9TMS |  | 2959 | MPI | 0,0588716 |
| UPSC_10400_GCTOF_Ath _RI_2971 | Unknown |  | 2971 |  | 0,0595218 |
| UPSC_10401_GCTOF_Ath _RI_2971 | Unknown |  | 2971 |  | 0,0523699 |
| UPSC_10402_GCTOF_Ath _RI_3115 | Digalactosylglycerol 9TMS^e^ |  | 3115 | MPI | 0,0885816 |
| UPSC_10403_GCTOF_Ath _RI_3133 | alpha-Tocopherol TMS |  | 3133 | UPSC | 0,0615668 |
| UPSC_10404_GCTOF_Ath _RI_3365 | Raffinose 11TMS |  | 3365 | UPSC | 0,0701336 |
| UPSC_10405_GCTOF_Ath _RI_3372 | Unknown | Saccharide | 3372 |  | 0,0691816 |
| UPSC_10406_GCTOF_Ath _RI_3567 | Unknown |  | 3567 |  | -0,0868403 |
| UPSC_10407_GCTOF_Ath _RI_3762 | Unknown |  | 3762 |  | 0,0694297 |
