# Supplementary material for: Improper excess light energy dissipation in Arabidopsis results in a metabolic reprogramming
Source: BMC Plant Biol. 2009 Jan 26;9:12. doi: 10.1186/1471-2229-9-12 (PMC2656510; doi:10.1186/1471-2229-9-12)
Supplement: Additional file 3 — The 23 genes with the highest probability for differential gene expression between npq4 and oePsbS. Positive p-values correspond to higher expression in oePsbS, and negative values to higher expression in npq4. [file 1471-2229-9-12-S3.doc]

Additional File 3: The 23 genes with the highest probability for differential gene expression *npq4* and oePsbS. Positive p-values correspond to higher expression in oePsbS, and negative values to higher expression in *npq4*.

AGI-code description M value *P* value

At2g34680 leucine-rich repeat family protein +5.62 0.000005

At5g03350 legume lectin family protein contains Pfam domain +2.33 0.00071

At3g19030 expressed protein contains similarity to phosphoserine +1.06 0.00071

At1g74950 expressed protein -1.00 0.00047

At1g34060 alliinase family protein contains Pfam profiles -1.01 0.00047

At4g19430 expressed protein -1.08 0.00073

At2g32150 haloacid dehalogenase-like hydrolase family protein -1.09 0.00042

At2g47180 galactinol synthase, putative similar to galactinol synthase -1.21 0.00026

At1g52720 expressed protein -1.23 0.00026

At2g34810 FAD-binding domain-containing protein similar to SP|P30986 -1.24 0.0007

At1g66390 myb family transcription factor, putative -1.27 0.0005

At2g21050 amino acid permease, putative similar to AUX1 -1.30 0.00022

? unknown protein -1.30 0.00053

At1g28330 dormancy-associated protein, putative (DRM1) -1.53 0.00053

At2g21130 peptidyl-prolyl *cis-trans* isomerase/cyclophilin (CYP2) -1.68 0.00005

At1g17420 lipoxygenase, putative similar to lipoxygenase -1.75 0.00042

At3g30320 hypothetical protein -1.81 0.00072

? unknown protein -2.05 0.00075

At4g15210 beta-amylase (BMY1) / 1,4-alpha-D-glucan maltohydrolase -2.10 0.00053

At4g08870 arginase, putative; similar to Swiss-Prot:P46637 arginase -2.21 0.00018

At4g18440 adenylosuccinate lyase, putative / adenylosuccinase -2.38 0.00053

At4g36950 pseudogene, similar to OSJNBa0042L16.2 -2.45 0.00026
